# Infrared Small-Target Segmentation Framework Based on Morphological Attention and Energy Core Loss

**DOI:** 10.3390/jimaging12050184

**Published:** 2026-04-24

**Authors:** Baoyu Zhu, Qunbo Lv, Yangyang Liu, Haoran Cao, Zheng Tan

**Affiliations:** 1 Aerospace Information Research Institute, Chinese Academy of Sciences, No. 9 Dengzhuang South Road, Haidian District, Beijing 100094, China; zhubaoyu20@mails.ucas.ac.cn (B.Z.); lvqunbo@aoe.ac.cn (Q.L.); liuyy@aircas.ac.cn (Y.L.); caohaoran24@mails.ucas.ac.cn (H.C.); 2 School of Optoelectronics, University of Chinese Academy of Sciences, No. 19(A) Yuquan Road, Shijingshan District, Beijing 100049, China; 3 Department of Key Laboratory of Computational Optical Imagine Technology, Chinese Academy of Sciences, No. 9 Dengzhuang South Road, Haidian District, Beijing 100094, China

**Keywords:** infrared imaging, object segmentation, small infrared targets, morphological attention, deep learning, U-Net

## Abstract

Infrared small-target segmentation (IRSTS) is crucial for a wide range of applications, including maritime search-and-rescue operations and intelligent traffic surveillance. However, current deep learning methods struggle with dynamic scale variations in infrared small targets, resulting in false detections and missed detections, alongside inadequate core localization accuracy. To address these challenges, we propose an infrared small-target segmentation framework founded on morphological attention and an energy core loss function, IRSTS_Unet. Specifically, we design a Dynamic Shape-adaptive Deformable Attention Module (DSDAM), which achieves parameterized feature extraction via “initial localization–offset deformation–precise sampling”. This approach enables the network to differentially focus on target cores and background cues to suppress clutter. To improve the efficiency of multi-scale feature aggregation, we embed the DSDAM within both the feature extraction and cross-layer fusion stages. Furthermore, we formulate a Core Energy-aware Core-Priority loss (CECP-Loss) function that incorporates the energy prior distribution of small targets, effectively counteracting the “core dilution” phenomenon endemic to conventional loss functions. Through extensive experiments on multiple public datasets, we demonstrate that IRSTS_U-Net outperforms state-of-the-art approaches in terms of both detection accuracy and robustness.

## 1. Introduction

Infrared small-target segmentation is a core task in the field of computer vision. It plays a fundamental supporting role in a wide range of real-world application scenarios, including maritime search-and-rescue operations, intelligent traffic surveillance, and forest fire prevention and early warning [1,2,3,4]. However, this task is plagued by numerous challenges arising from the inherent properties of the targets. Confined by imaging distance and the physical constraints of imaging devices, infrared small targets often span only a few pixels and lack prominent discriminative features such as texture and contour. Moreover, variations in capture distance introduce dynamic changes in target scale and morphology. Additionally, these targets are prone to being obscured by complex background clutter—including cloud layers, ground vegetation, and light interference—all of which collectively exacerbate the prominence of false detections and missed detections [5,6,7].

Early research predominantly hinged on conventional paradigms, including filter-based background suppression strategies [8], local-contrast-driven target enhancement algorithms [9,10,11], and low-rank–sparse decomposition-powered feature separation techniques [12,13,14]. While these methods can exhibit some efficacy in simple scenarios, they struggle to adapt to complex and dynamic real-world environments due to their excessive reliance on hand-crafted feature templates. When background clutter fluctuates violently or target signals become extremely faint, their detection performance undergoes notable degradation [3].

Alongside the advancement of deep learning technology, data-driven deep learning methods have gradually emerged as the dominant research paradigm in IRSTS [15,16]. Researchers in the field have drawn on classic frameworks for semantic segmentation and object detection to put forward a suite of improved models, for instance, nesting U-Net architectures to enable multi-scale feature aggregation [17,18], or crafting specialized modules to boost the propagation of small-target features [2,19]. These methods leverage an end-to-end training paradigm to automatically learn features and have yielded modest gains in enhancing robustness under complex scenarios. However, current research still suffers from two pivotal limitations that constrain further breakthroughs in performance in IRSTS:

1. Limited Scene Adaptability of Dynamic Attention Mechanisms

Recent studies have extensively explored adaptive, scale-aware, and deformable attention mechanisms for infrared vision tasks, effectively enhancing the ability to extract features from objects of varying scales. However, most of these methods are designed for general vision tasks and lack specific optimizations tailored to the inherent characteristics of small infrared targets (ultra-small size, weak edges, and dynamic scale changes). Static sampling grids and global context modeling remain widely adopted, but they are prone to introducing background noise, leading to feature responses being easily overwhelmed by noise in scenes where small targets are highly similar to the background [20]. Meanwhile, Transformer-based attention methods still face challenges such as high computational complexity, slow convergence, and a high risk of overfitting when processing high-resolution infrared images containing minute targets.

2. Insufficient Property Sensitivity of Loss Functions

Mainstream loss functions (e.g., IoU loss, GIoU loss) lack targeted adaptation to target scale variations and positional deviations, which tends to reduce localization accuracy during the detection of small targets [21]. These loss functions fail to account for the inherent distribution characteristics of infrared small targets—strong central energy and blurred edges—and employ identical loss weights for both core and edge regions, often resulting in core localization offsets or excessive edge segmentation.

To surmount the twin bottlenecks of generic feature extraction and core-agnostic loss guidance in IRSTS, we propose an infrared small-target segmentation framework grounded in morphological attention and energy core loss and design a Dynamic Shape-adaptive Deformable Attention Module (DSDAM), comprising three-stage feature extraction: “initial position design–offset-driven deformation–shape-aware feature aggregation”. This model overcomes the fixed sampling limitations of traditional attention mechanisms, facilitates the flexible parameterized configuration of attention sampling, and can be seamlessly integrated into the feature extraction and fusion stages of mainstream detection frameworks. It can also differentiate and focus on the target’s core region and key background cues, strengthening central feature responses by leveraging the intrinsic “strong core–weak edge” signature of small targets and dynamically suppressing irrelevant clutter based on the complexity of the background. This design fully exploits the correlative information between the background and target to improve feature discriminability. Embedding the DSDAM into the feature extraction and cross-layer fusion stages of the framework, we strengthen the target orientation of cross-layer feature fusion by dynamically adjusting attention sampling regions. We also utilize the morphological adaptability of the DSDAM to refine the attention-driven aggregation of multi-scale features before they are fed to the detection head. While preserving fine-grained features of small targets, this design utilizes contextual information efficiently to support target identification—markedly enhancing both the accuracy of infrared small-target segmentation and its robustness in complex scenarios.

To address the issue of insufficient property sensitivity in loss functions, this study proposes Core Energy-Aware Core-Priority loss (CECP-Loss), which comprises a two-step lightweight design: “energy-aware weight generation → core-priority loss calculation”. Without relying on additional auxiliary modules, this loss function can incorporate the “strong core–weak edge” energy prior distribution of infrared small targets into the training optimization process, effectively solving the problem of “core contribution dilution by edge regions” inherent in conventional loss functions.

Specific contributions are as follows:

1. We propose a novel Dynamic Shape-adaptive Deformable Attention Module (DSDAM). Based on the strategy of “initial position design → offset-driven deformation → shape-aware feature aggregation”, this module realizes flexible parameterized sampling, which enables the network to pay differentiated attention to the target core region and key background cues. It enhances the central feature response of small targets while suppressing background clutter.

2. A modified U-Net detection framework is developed, which incorporates the DSDAM into the feature extraction and fusion stages. By dynamically adjusting attention sampling, this framework enhances the target specificity of cross-layer fusion; prior to the prediction head, it further refines the aggregation of multi-scale features while preserving fine-grained details of small targets and context-assisted target discrimination.

3. Core Energy-Aware Core-Priority loss (CECP-Loss) is developed, which incorporates the energy prior distribution of infrared small targets with a two-step lightweight design, “energy-aware weight generation–core-priority loss calculation”, thereby addressing the issue of “core contribution dilution” in conventional loss functions.

4. We conduct extensive experiments on multiple public datasets, where the proposed method outperforms existing state-of-the-art approaches in both detection accuracy and robustness. This validates the effectiveness of the adaptive attention mechanism and energy core loss, thereby providing a better solution for infrared small-target segmentation.

## 2. Related Work

Conventional single-frame detection algorithms can be broadly grouped into three paradigms: those based on background consistency, the human visual system, and mathematical optimization models. Methods predicated on background consistency assume that the background in infrared small-target images tends to exhibit similar characteristics, while the presence of small targets disrupts the inherent correlation of the background [22]. Such methods achieve target segmentation via background subtraction; however, in complex background scenarios, they are susceptible to noise interference, resulting in false alarms. Methods inspired by the human visual system’s “selective attention” use saliency to identify targets from the background [23,24,25]. Such methods are typically dependent on human prior assumptions and exhibit low robustness under complex conditions. Finally, some models leverage the properties of low-rank background and sparse targets in image data to establish mathematical optimization models, thereby distinguishing between the background and targets [26,27,28,29]. These methods offer high accuracy but exhibit poor real-time performance [30].

Recently, deep learning methods have developed rapidly in the field of infrared target detection and segmentation. Given that the feature characteristics of infrared small targets are highly compatible with the operational logic of convolutional neural networks (CNNs), CNN-based methods possess inherent advantages. MTMLNet [31] introduces a Multi-level Feature Aggregation (MFA) module that concurrently captures features across different gradients and receptive fields, boosting both detection and segmentation by using heterogeneous supervisory signals. IDNA-Unet [32] employs a Dense Nested Interaction Module (DNIM) as its feature extractor, progressively fusing features within a U-Net backbone to retain the fine-grained signatures and precise localization cues of diminutive targets. EGPNet [33] utilizes a Multi-scale Feature Progressive Fusion (MFPF) encoder to harvest features, enriching semantic cues and contextual coherence; this is coupled with an Edge-Guided Image Refinement Module (EIRM), which preserves the target’s shape integrity. MFEU-Net [18] builds upon U-Net and presents a dedicated network for dim-infrared small-target detection; its encoder–decoder stack of Residual U-blocks and Inception modules harvests rich multi-scale features, enabling accurate localization in cluttered scenes. PConv [34] tackles the mismatch between standard convolutions and the Gaussian-like signatures of IR small targets by proposing the Pinwheel Convolution (PConv) module. Its specially designed kernels amplify target–background contrast while markedly enlarging the receptive field. IR-ADMDet [35] employs a dual-path hybrid feature extractor that synergizes local residual learning with global context modeling, reinforcing faint target signatures and simultaneously suppressing distractors.

To enhance detection accuracy, Transformer-based methods have been introduced into the field of infrared small-target detection. Rkformer [36] uses a random connection attention module, achieving a balance between semantic extraction and detail preservation in infrared small-target detection. To balance local and global dependencies, CNN and Transformer components are often integrated. MTU-Net [37] utilizes a hybrid encoder combining a Vision Transformer (ViT) and CNN to extract multi-level features. RDIAN [38] adopts convolutional layers with different receptive fields to capture multi-local target features with the aim of enhancing feature diversity, leveraging a multi-directional guided attention mechanism to strengthen target feature representation. APTNet [39] utilizes dual residual attention blocks and adaptive partial Transformer modules to enhance the integration of contextual information, thereby enabling the accurate detection of small targets in complex scenarios and improving detection performance. To address the difficulty in identifying infrared small targets in complex scenarios, STASPPNet [40] combines a Swin Transformer with a multi-scale dilated spatial pyramid pooling module to improve both feature representation and target detection accuracy. However, Transformers suffer from high computational complexity, stringent hardware resource requirements, and low efficiency when processing high-resolution images, limiting their application in practical scenarios [41].

## 3. Methodology

**Figure 1:**
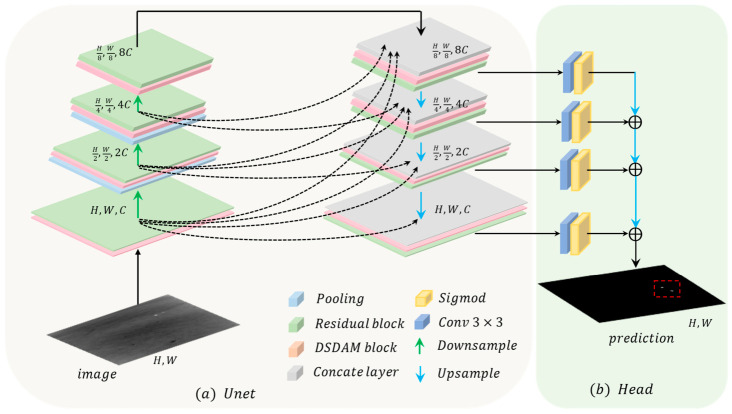
Structure Diagram of Infrared Target Segmentation Model. (**a**) Schematic diagram of the U-Net architecture used in this method. (**b**) Schematic diagram of the detection head structure used in this method.

Figure 1 presents a structure diagram of the infrared small-target segmentation framework based on morphological attention and energy core loss. Based on the traditional U-Net architecture and with reference to [42], this study employs a four-layer deep U-Net architecture, incorporating a shape-deformable attention module into the feature extraction and fusion stages to accomplish multi-scale feature fusion and target prediction. Through the synergy of its components, the overall framework preserves small-target details, leverages contextual information, and thereby enhances the accuracy and robustness of infrared small-target segmentation.

In the feature extraction stage, the network can dynamically adjust the attention sampling regions when extracting features at different levels by embedding a dynamic deformable attention module into each layer of the encoder. For low-level features, the module focuses more on capturing fine-grained details of small targets, strengthening sampling on the target’s core region to preserve the fine-grained features of small targets. For high-level features, the module instead focuses on the overall relationship between the target and the background, leveraging key background cues to assist in target identification.

In the feature fusion stage, cross-layer feature fusion is a key advantage of the U-Net architecture, and integrating the shape-deformable attention module further enhances the target specificity of this fusion. Based on the size and position of targets in features of different scales, the module dynamically adjusts attention weights, ensuring that features from different levels fulfill their respective roles during the fusion process. Fine-grained details of small targets in low-level features and contextual information in high-level features are effectively integrated, thereby enhancing the quality of fused features.

Attention aggregation is performed on multi-scale features before they reach the head prediction module by leveraging the shape-adaptive properties of the shape-deformable attention module. By assigning various attention weights to features of different scales, features considered critical for the detection task are emphasized, and the final features input to the prediction module are optimized, thereby enhancing the accuracy and robustness of target segmentation. Additionally, while preserving fine-grained details of small targets, the framework fully utilizes contextual information to assist in discrimination, effectively addressing the issue of small targets being easily overwhelmed by the background.

### 3.1. Dynamic Shape-Adaptive Deformable Attention Module (DSDAM)

**Figure 2:**
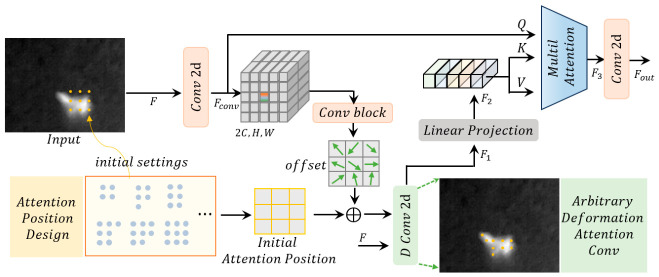
Structural Diagram of the Dynamic Shape-adaptive Deformable Attention Module (DSDAM).

The Dynamic Shape-adaptive Deformable Attention Module (DSDAM) serves as the core component for feature extraction in this framework, employing a strategy of “initial position design–offset-driven deformation–shape-aware feature aggregation” to break through the inherent limitations of traditional attention mechanisms. Its structural diagram is shown in Figure 2. The module takes as input a feature map $F\in R^{H\times W\times C}$ (where H and W are the height and width of the feature map, and C is the number of channels) and outputs the feature $F_{\mathrm{out}}$ processed by multi-head attention and convolution, whose dimensions are consistent with those of the input feature.

The Dynamic Shape-adaptive Deformable Attention Module (DSDAM) realizes adaptive feature extraction for infrared small targets through a cohesive process of “initial position design → offset-driven deformation → shape-aware feature aggregation”. The DSDAM can dynamically adjust the [sampling] density via a configurable factor, r; during the offset stage, it incorporates an energy map to guide sampling points to converge on the target core. This module outperforms the fixed logic of static attention and the local dynamics of low-adaptability attention by a significant margin.

The detailed design of the DSDAM proposed in this paper is as follows.

For the input feature map $F\in R^{H\times W\times C}$ (where H and W are the height and width of the feature map, and C is the number of channels), the module first generates the initial set of sampling points using a configurable grid strategy:

(1) $$P_{\mathrm{init}}=\{p_{i}{\}}_{i=1}^{N_{s}}$$

where $N_{s}$ (the total number of sampling points) is calculated as $N_{s}=H_{G}\times W_{G}$; $H_{G}$ and $W_{G}$ are defined as $H_{G}=H/r,\;W_{G}=W/r$, with their exact values determined by the floor function via $H_{G}=\left\lfloor \frac{H}{r}\right\rfloor ,\;W_{G}=\left\lfloor \frac{W}{r}\right\rfloor$ (where ⌊⋅⌋ denotes the floor function, which rounds down to the nearest integer); and r is an adjustable downsampling factor.

The spatial coordinates of the initial sampling points $p_{k,l}$ (where $k\in \left[0,H_{G}-1\right]$ and $l\in \left[0,W_{G}-1\right]$) are generated using the following formula:

(2) $$p_{k,l}=\left(l\cdot r+\frac{r}{2},k\cdot r+\frac{r}{2}\right)$$

where $\left(l\cdot r+\frac{r}{2}\right)\;$ and $\left(k\cdot r+\frac{r}{2}\right)$ correspond to the pixel coordinates of the sampling points in the horizontal and vertical directions of the feature map, respectively. This ensures that each sampling point is located at the center of the corresponding grid cell, thereby enhancing the representativeness of local features.

The core principle for the assignment of r is dynamic adaptation based on the scale characteristics of infrared small targets. This design endows the module with scalability: reducing r can increase the sampling density to capture fine-grained features of small targets, while increasing r can decrease the density to balance the computational efficiency of large target/global shape modeling.

Based on the convolutional feature map $F_{\mathrm{conv}}\in R^{2C\times H\times W}$ obtained by convolving the input feature, F, the spatial offset is learned through a lightweight convolutional branch:

(3) $$\Delta P={\mathrm{DWConv}}_{2}d\left(\mathrm{ReLU}\left({\mathrm{Conv}}_{2}d\left(F_{\mathrm{conv}}\right)\right)\right)$$

where ${\mathrm{DWConv}}_{2}d$ denotes depthwise separable convolution. These offsets possess the “target-and-background-aware” property: for small targets with strong center and weak edge responses, the offsets guide sampling points to gather toward the core to strengthen center responses, and for complex background regions, the offsets drive sampling points away from clutter to suppress interference. Finally, the initial positions are fused with the offsets to obtain the deformed attention positions (D Conv 2d).

(4) $$P_{D\;\mathrm{Conv}}=P_{\mathrm{init}}+\Delta P$$

The position after deformation guides the deformable convolution (D Conv 2d) to sample the input feature, F, resulting in the deformed feature, $F_{1}$, and achieving dynamic adaptation to the target shape. Feature $F_{1}$ undergoes linear projection to obtain the projected feature, $F_{2}$, which is then used to generate the key (K), and value (V). Query (Q) is generated from the input feature F.

Subsequently, the multi-head attention module performs feature aggregation through fusion, and its core computation can be simplified as follows:

(5) $$F_{3}=Softmax\left(\frac{Q\cdot K^{⊤}}{\sqrt{d}}\right)\cdot V$$

where Softmax denotes the Softmax activation function, $K^{⊤}$ denotes the transpose of K. First, we compute the dot product similarity between query Q and key K and then divide it by the feature dimension, $\sqrt{d}$, to avoid numerical instability caused by excessively large dot product results. Next, we convert the similarity into attention weights via the activation function, $\sqrt{d}$. Finally, we perform a weighted summation on the value vector, V, using these weights to obtain the aggregated feature, which is then output ($F_{\mathrm{out}}$) after convolution.

The DSDAM achieves adaptive feature extraction for infrared small targets of different scales and shapes through its cohesive process.

### 3.2. Core Energy-Aware Core-Priority Loss (CECP-Loss)

To address the insufficient sensitivity of loss functions to target characteristics in infrared small-target segmentation, this study proposes Core Energy-Aware Core-Priority loss (CECP-Loss), which consists of two cascaded components: “energy-aware spatial weight generation” and “core fitting loss calculation.” Neither component relies on external modules nor requires redundant computations, enabling them to be directly embedded into mainstream segmentation frameworks for end-to-end training.

For the generation of Energy-Aware Spatial Weight $\omega_{\mathrm{spatial}}\left(i\right)$, we first segment the target foreground region, $\Omega_{\mathrm{fg}},$ using the ground truth label g (where $\left(g_{i}=1\right)$ denotes a foreground pixel and $\left(g_{i}=0\right)$ denotes a background pixel) and then compute its geometric center $\left(x_{c},y_{c}\right)$. Compared to the complex energy-weighted center, the geometric center not only ensures localization accuracy but also significantly simplifies computation, striking a balance between performance and efficiency:

(6) $$x_{c}=\frac{1}{S}\sum_{i\in \Omega_{\mathrm{fg}}}x_{i}\;$$

(7) $$y_{c}=\frac{1}{S}\sum_{i\in \Omega_{\mathrm{fg}}}y_{i}$$

where S represents the total number of foreground pixels, and $\left(x_{i},y_{i}\right)$ denotes the image coordinates of pixel i.

We use a Gaussian decay function to map $d_{i}$ to spatial weight $\omega_{\mathrm{spatial}}\left(i\right)$, which simulates the energy decay law of infrared targets characterized by a stronger core and weaker edges:

(8) $$\omega_{\mathrm{spatial}}\left(i\right)=\exp\left(-\frac{d_{i}^{2}}{2\sigma^{2}}\right)$$

where σ denotes the adaptive standard deviation, which is determined by maximum target radius $R_{\max}$ (i.e., the maximum distance from the target boundary pixels to the center) and set as $\sigma =0.3R_{\max}\;$. $d_{i}$ represents the Euclidean distance from pixel i to the center $\left(x_{c},y_{c}\right)$, calculated as $\;d_{i}=\sqrt{{\left(x_{i}-x_{c}\right)}^{2}+{\left(y_{i}-y_{c}\right)}^{2}}$, which measures the spatial correlation between the pixel and target core.

The generated spatial weight, $\omega_{\mathrm{spatial}}\left(i\right)$, is embedded into the base Dice Loss [43], and a core-focused loss is constructed in a single step, forcing the model to prioritize the optimization of the core region. The formula is as follows:

(9) $$L_{CECP}=1-\frac{\sum_{i=1}^{H\times W}w_{spatial}\left(i\right)\cdot p_{i}\cdot g_{i}+eps}{\sum_{i=1}^{H\times W}w_{spatial}\left(i\right)\cdot \left(p_{i}+g_{i}\right)+eps}$$

where $p_{i}$ denotes the foreground prediction probability of pixel i output by the model (where $p_{i}\in \left[0,1\right]$); $eps=10^{-6}$ is a smoothing term, used to avoid numerical anomalies caused by a zero denominator; the high weight $\omega_{\mathrm{spatial}}\left(i\right)\;$ assigned to the core region renders the loss value more sensitive to the prediction errors of core pixels.

## 4. Experiments

### 4.1. Datasets

IRSTD-1K [44]: This dataset contains 1001 high-resolution (512 × 512 pixels) real infrared images, covering complex backgrounds such as urban areas, forests, and sea surfaces. Accurate bounding boxes and semantic masks are annotated for over 1500 small targets. As the first public benchmark for infrared small-target segmentation, IRSTD-1K is widely used to verify the robustness of deep learning models.

NUDT-SIRST [45]: Focusing on small-target segmentation in complex environments, this dataset includes 1327 real infrared images (256 × 256 pixels), covering scenarios such as urban night scenes, ships at sea, and unmanned aerial vehicles (UAVs) flying through clouds. The average target size is only 9 × 9 pixels, and the signal-to-noise ratio (SNR) is as low as 0.3 dB.

### 4.2. Evaluation Metrics

Model performance is evaluated using three metrics: Intersection over Union (IoU), Probability of Detection (Pd), and False Alarm Rate (Fa).

IoU is the core metric for measuring the localization accuracy of infrared small targets, with a value range of [0,1]. It focuses on spatial localization accuracy and is sensitive to the geometric deformation of small targets.

(10) $$\mathrm{IoU}=\frac{\left|A\cap B\right|}{\left|A\cup B\right|}$$

where A refers to the predicted target region output by the model and B denotes the manually annotated ground truth target region. When IoU approaches 1, this indicates that the model accurately captures the position and size of infrared small targets. When IoU approaches 0, it means the predicted region does not overlap with the ground truth target, implying severe localization deviation or false detection. IoU is often used as the criterion for determining “correct detection”. In this study, IoU≥0.5 is defined as a valid detection, which serves as the basis for subsequent calculations of Pd and Fa.

Pd is used to measure the detection capability of infrared small targets, with a value range of [0,1], and it is also known as Recall:

(11) $$\mathrm{Pd}=\frac{N_{\mathrm{correct}}}{N_{\mathrm{total}}}$$

where $N_{\mathrm{correct}}$ denotes the number of real targets that are correctly detected, which must satisfy the condition that “the IoU between the predicted region and the real target is ≥the preset threshold”, and $N_{\mathrm{total}}$ represents the total number of all real targets in the infrared image/sequence.

Fa stands for False Alarm Rate (Fa for short), and its calculation formula is as follows:

(12) $$\mathrm{Fa}=\frac{N}{P_{\mathrm{total}}}$$

where N denotes the number of false alarm targets and $P_{\mathrm{total}}$ represents the total number of predictions output by the model.

### 4.3. Experimental Details

All experiments in this study were conducted on a computer equipped with NVIDIA RTX A5000 (24 GB VRAM × 4 cards), paired with an Intel Xeon Gold 5320 CPU and 128 GB RAM. The system runs 64-bit Ubuntu, and the deep learning framework used is PyTorch 2.1. The model adopts the Adam optimization algorithm [46], with the initial learning rate set to 0.1. The linear decay of the learning rate starts at the 100th epoch, reducing it to 10^−6^, and the total training process consists of 300 epochs. The training set and test set are divided in a ratio of 8:2.

All experiments in this work strictly adhere to the widely accepted benchmark testing protocols in the infrared small target segmentation field. We employ the official train/test splits defined by the dataset authors, fix all random seeds throughout the training and inference process, and maintain consistent training hyperparameters and hardware environment across all comparative experiments.

### 4.4. Comparative Experimental Results

To verify the effectiveness of the proposed IRSTS_Unet framework in the infrared small-target segmentation and detection task, this section selects advanced IRSTS methods that have emerged in recent years—including ACLnet [47], ISNet [44], AGPCNet [48], DNA-Net [45], MSHNet [21], and EGPNet [33]—as baselines, conducting comparative experiments on two public standard datasets: IRSTD-1K and NUDT-SIRST.

The quantitative experimental results on the NUDT-SIRST dataset are shown in Table 1. The proposed IRSTS_Unet exhibits more prominent advantages on this dataset, ranking first in all three metrics, which fully verifies its adaptability to ultra-small targets.

The IRSTS-Unet proposed in this paper also maintains highly competitive inference efficiency, achieving a frame rate of 63.8 FPS under a unified test standard, and realizes a favorable trade-off between segmentation accuracy and computational efficiency.

The IRSTD-1K dataset contains infrared images of complex scenarios such as cloud occlusion, vegetation interference, and light clutter. It has a large range of target sizes, placing high demands on the model’s scene adaptability and small-target capture capability. As shown in Table 2, the quantitative experimental results indicate that the proposed IRSTS_Unet demonstrates excellent performance on all three metrics, outperforming most baseline methods.

The overall Probability of Detection (Pd) of the IRSTS_Unet on the IRSTD-1K test set reaches 94.1%, while there are significant differences in detection performance for targets of different sizes: for ultra-small targets smaller than 4 pixels, accounting for 8.75% of the total targets, the Pd is only 76.07%; for mainstream size targets of 4–36 pixels, accounting for 66.67% of the total targets, the Pd reaches 94.76%; and for larger targets larger than 36 pixels, accounting for 24.58% of the total targets, the Pd is as high as 97.78%.

**Figure 3:**
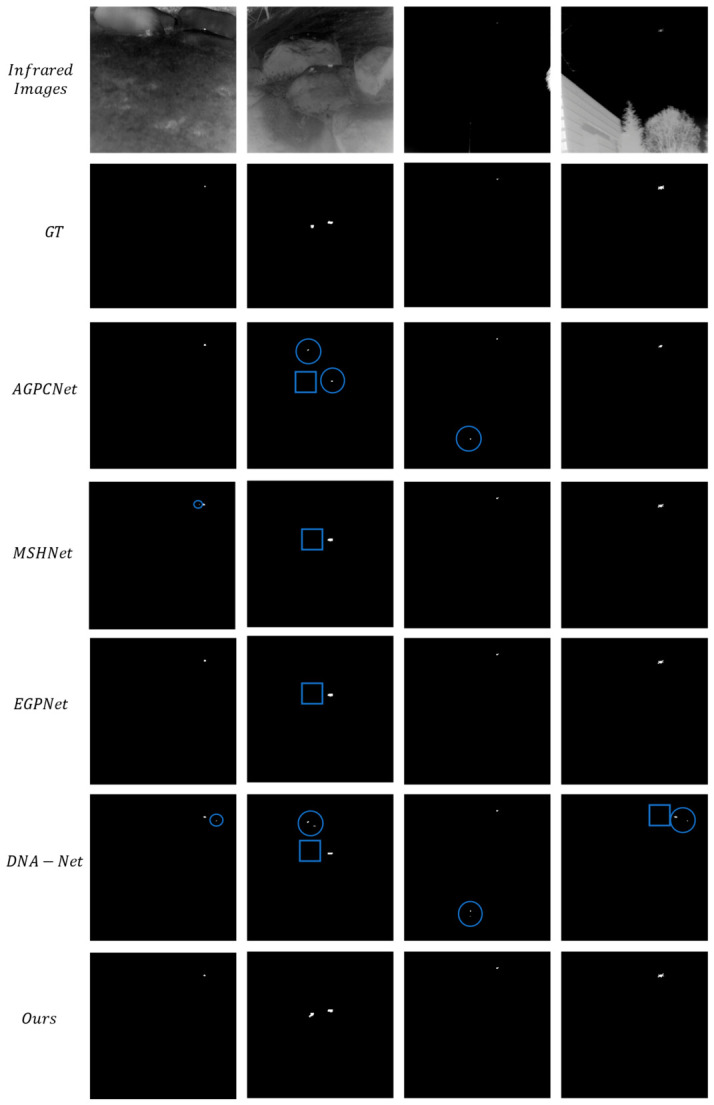
Qualitative comparison results on the IRSTD-1K dataset.

Figure 3 reveals the results of the qualitative comparative experiment. The first row presents the original infrared images, covering typical application scenarios such as sea surfaces, complex terrain, and nighttime scenes. The second row shows the corresponding ground truth (GT), with round boxes denoting false positives and rectangular boxes representing false negatives. The third to seventh rows sequentially display the detection results of AGPCNet, MSHNet, EGPNet, DNA-Net, and the proposed method in this study. AGPCNet fails to detect some small targets with weak signals, an issue that also besets MSHNet in certain scenarios; the results of EGPNet are close to GT values but are still hindered by minor localization deviations and missed detections. Although DNA-Net can detect targets, it suffers from both false alarms and missed detections. By contrast, the detection results of our proposed method are better in terms of target integrity, localization accuracy, and background clutter suppression: it not only accurately captures the core regions and edge details of small targets but also effectively avoids false and missed detections caused by background clutter. The proposed method’s results more closely match the ground truth values, which verifies its effectiveness in complex scenarios.

**Figure 4:**
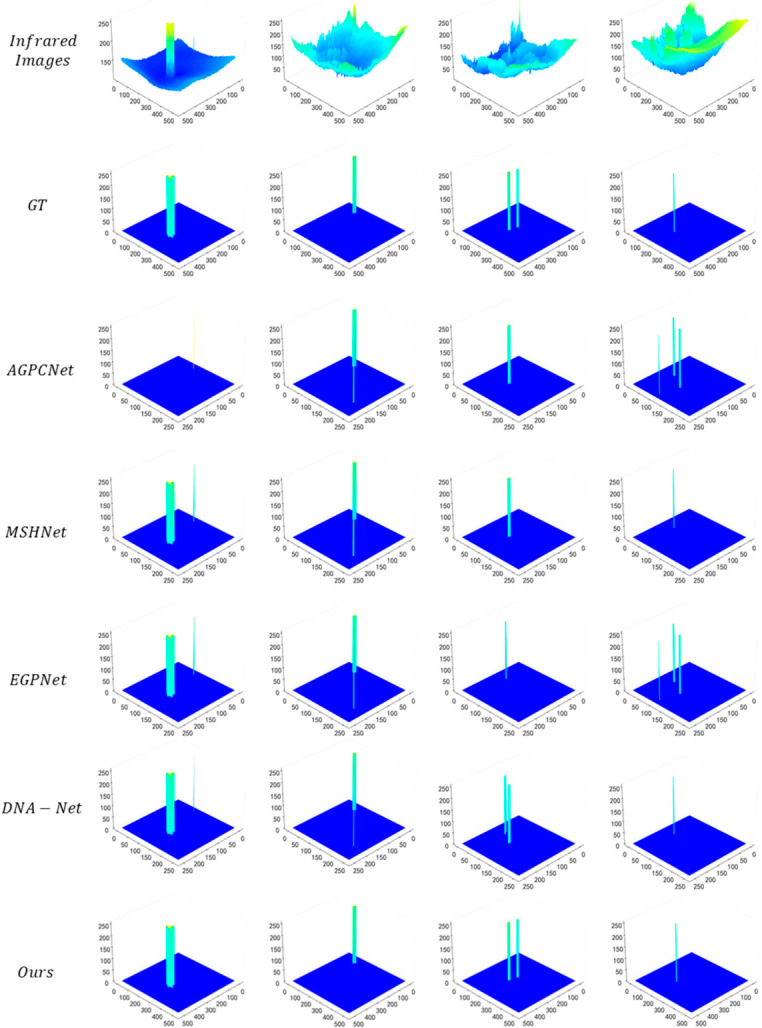
Three-dimensional visualization of detection results from various methods on the IRSTD-1K dataset.

The 3D visualization comparison of detection results from various methods is shown in Figure 4. The first row, Infrared Images, presents the 3D energy distribution of original infrared images, where obvious energy aliasing exists between targets and complex backgrounds. In the second row (GT), infrared small targets exhibit concentrated and prominent high-energy spikes, while the background maintains a low-energy stable state, providing a benchmark reference for detection results. AGPCNet fails to sufficiently capture the high-energy cores of some targets, leading to less prominent target spikes. Methods such as MSHNet and EGPNet generate false high-energy peaks in background regions, suffering from clutter false detection. DNA-Net shows a large deviation in spike morphology from GT in multi-target scenarios. In contrast, the 3D distribution of our method highly conforms to GT: the high-energy spikes of targets accurately match the ground truth in terms of morphology, position, and energy intensity, and the background regions remain in a low-energy stable state. This not only fully restores the energy characteristic of infrared small targets (strong in the center and weak at the edges) but also effectively suppresses the interference of background clutter, intuitively verifying the capability of our method to accurately capture the core energy of targets.

**Figure 5:**
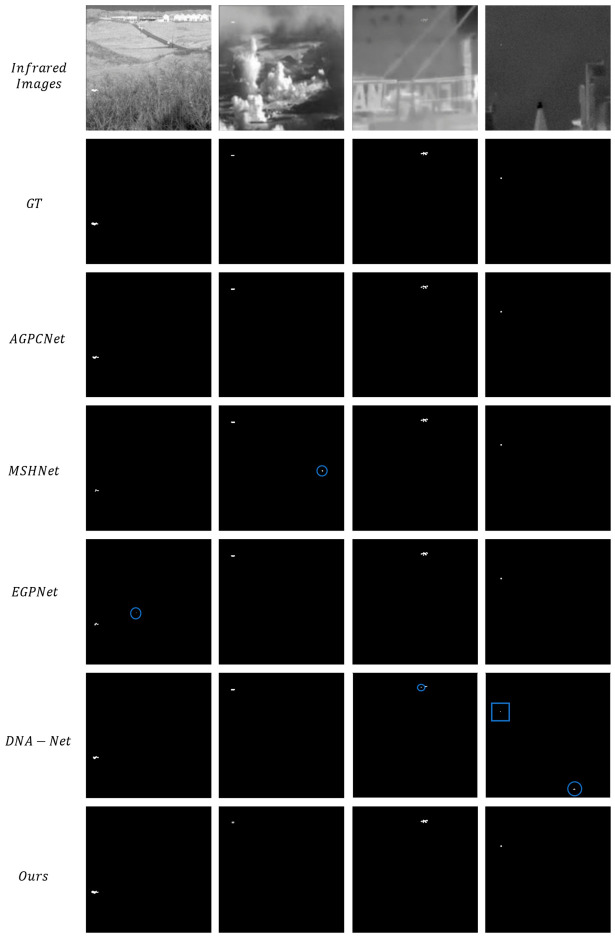
Qualitative comparison results on the NUDT-SIRST dataset.

**Figure 6:**
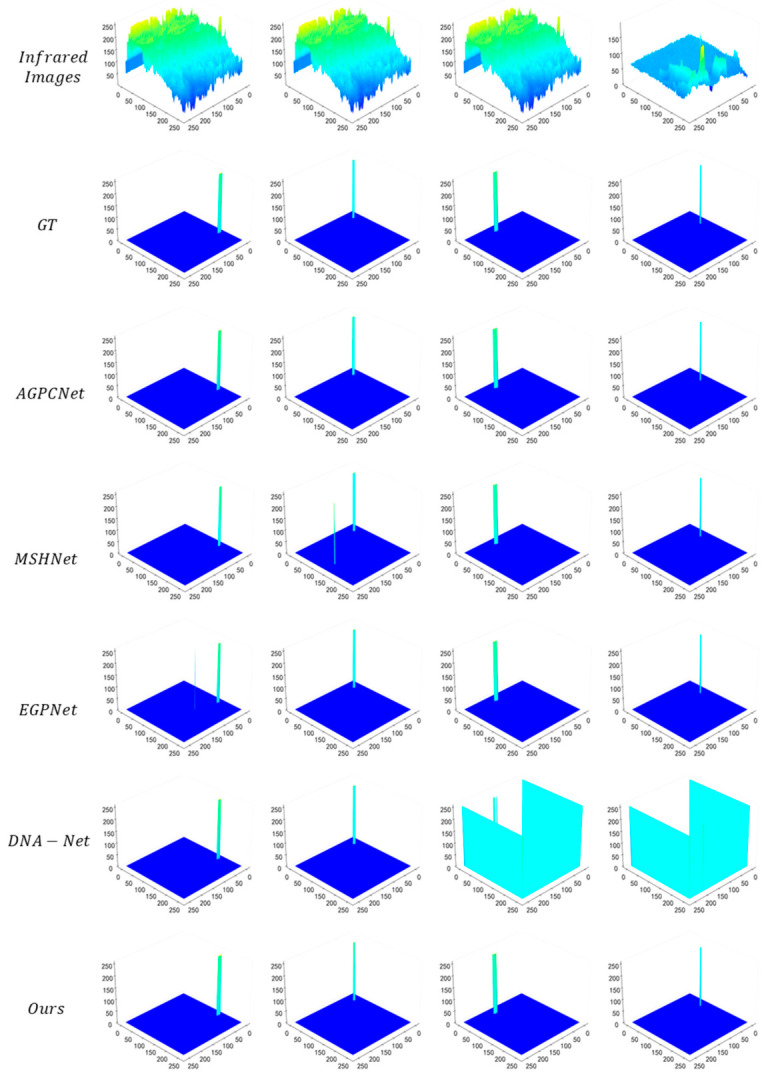
Three-dimensional visualization of detection results from various methods on the NUDT-SIRST dataset.

The experimental results from the qualitative comparison tests on the NUDT-SIRST dataset are shown in Figure 5 and Figure 6, which show that the proposed method exhibits higher consistency with the ground truth labels.

**Figure 7:**
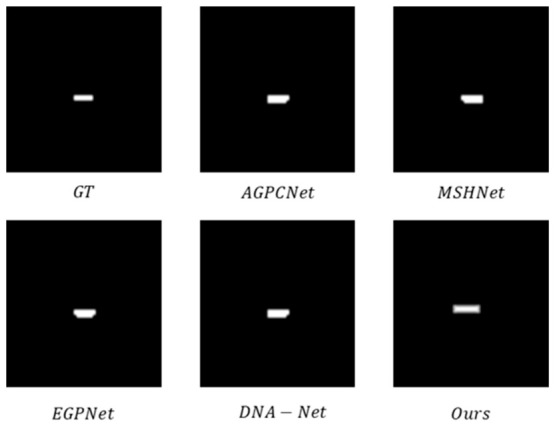
Comparative diagram of the detected regular shape.

**Figure 8:**
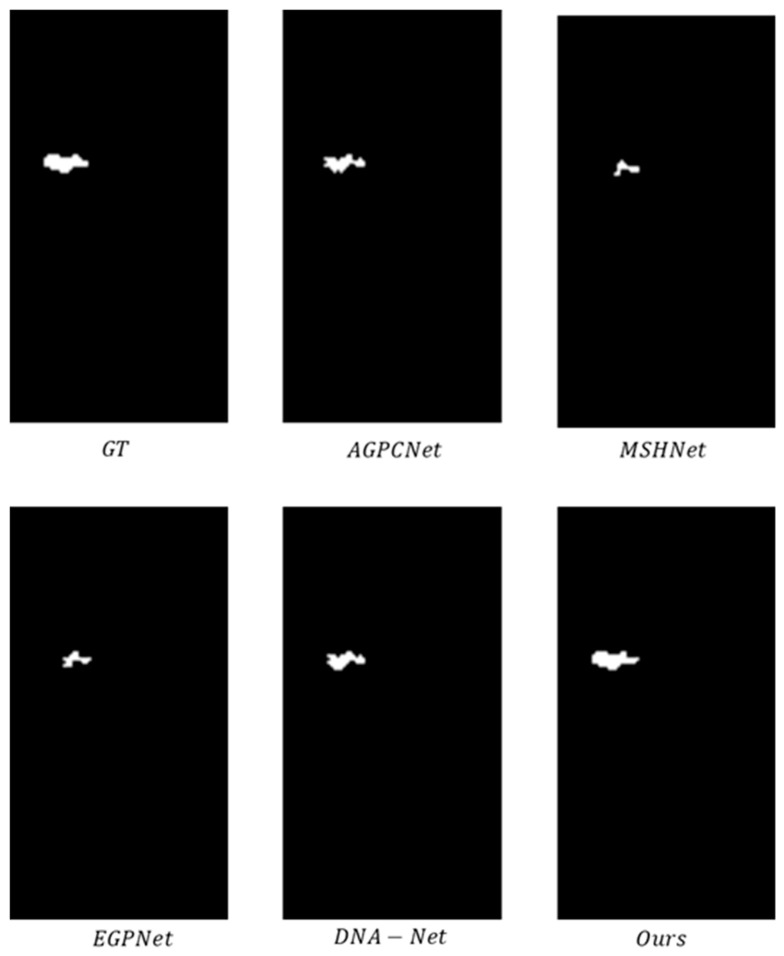
Comparative diagram of results of complex shape detection.

Figure 7 and Figure 8 shows a morphological comparison of the output results from various methods on the IRSTD-1K dataset, which intuitively verifies the morphological accuracy of the detection results. Figure 7 depicts targets with regular shapes: the GT (ground truth) presents small targets with compact and regular morphology, and our method accurately restores the complex contour of these targets, achieving high consistency with the GT in both shape integrity and detail fidelity. Figure 8 displays targets with complex shapes: the ground truth (GT) shows the morphology of targets with fine-grained details. Other methods introduce spurious edge artifacts or induce morphological distortion, weakening the morphological distinguishability of the target. In contrast, our method not only completely preserves the regular shape of the target but also maintains clear and sharp boundaries, fully demonstrating its capacity to accurately detect target morphology across different scenarios.

To further validate the cross-scene generalization capability of our proposed method, we have supplemented additional cross-dataset experiments. All training hyperparameters and preprocessing pipelines are kept fully consistent with the aforementioned comparative experiments, with no dataset-specific tuning performed for the target test set. The model is trained on the training set of NUDT-SIRST and tested on the test set of IRSTD-1K, with the results shown in Table 3. The experimental results show that our proposed IRSTS_Unet significantly outperforms all baseline methods on all key metrics in the cross-distribution test, which fully demonstrates the excellent cross-scene generalization capability of our method and confirms that there is no overfitting to a single dataset.

**Figure 9:**
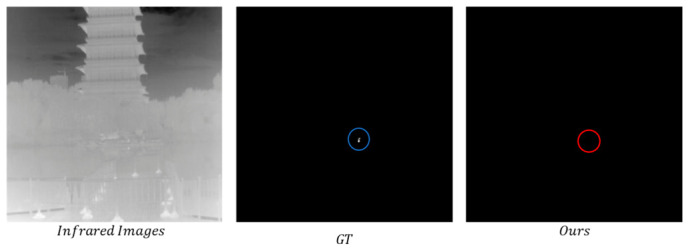
Missed detection case of small targets in an ultra-low signal-to-noise ratio scene.

Figure 9 shows a missed detection case. The image sample is from the IRSTD-1K dataset, corresponding to a complex urban night background scene. The ground truth targets are two spatially adjacent ultra-small infrared targets, each of which only occupies 2 × 3–3 × 3 pixels. The effective energy of the targets is completely submerged in the background clutter, and the proposed method fails to achieve effective detection at the corresponding position, resulting in missed detection.

### 4.5. Ablation Study

Ablation experiments conducted on the IRSTD-1K dataset verify the effectiveness and generality of the Dynamic Shape-Adaptive Deformable Attention Module (DSDAM) and the Energy-Aware Core-Priority Loss (CECP-Loss).

As shown in Table 4, when DSDAM is removed from IRSTS_Unet and replaced with conventional convolution (IRSTS_Unet-DSDAM + CECP-Loss), the model performance declines significantly: the Intersection over Union (IoU) decreases from 67.56% to 54.31%, the Probability of Detection (Pd) drops from 94.10% to 75.24%, and the False Alarm rate (Fa) rises from 15.02% to 30.84%. This indicates that DSDAM, through dynamic sampling and shape adaptation, is crucial for improving the accuracy of target feature extraction and reducing background interference.

As presented in Table 5, compared with the model using the traditional Dice loss, IRSTS-Unet with the integrated CECP-Loss achieves improvements in all three metrics. This proves that the design of CECP-Loss for “core energy priority optimization” of infrared small targets can effectively enhance target detection capability and suppress false alarms.

As demonstrated in Table 6, after migrating CECP-Loss to the MSHNet model, its performance is slightly improved compared with the original MSHNet. This further illustrates that CECP-Loss is not only compatible with the proposed IRSTS-Unet but also has a performance optimization effect on other infrared small target segmentation models, showing strong generality.

### 4.6. Analysis of Advantage Mechanisms

IRSTS comprehensively outperforms mainstream competing approaches in both detection accuracy (IoU, Pd) and robustness (Fa) on the IRSTD-1K and NUDT-SIRST datasets (Table 1 and Table 2). This superiority stems from the synergistic design of the DSDAM and CECP-Loss, which precisely addresses two key bottlenecks of traditional methods in dynamic feature extraction and core-focused optimization.

1. Dynamic Morphology Adaptation Mechanism Breaks the Limitations of Static Sampling

The attention mechanisms of mainstream competing approaches have inherent flaws: methods such as AGPCNet rely on fixed sampling patterns and cannot adjust the region of interest according to target scale and background complexity, leading to the smothering of small target features by background clutter (e.g., missed detections of AGPCNet and localization errors of MSHNet in Figure 3). The DSDAM in this work achieves parametric flexible sampling via the strategy of “initial localization—offset-driven deformation—morphology-aware aggregation”: the configurable factor r can dynamically adapt to target scales, and the offset is guided by the energy map, enabling sampling points to actively converge on the target core and move away from background clutter (as shown in the 3D energy maps in Figure 4, IRSTS yields more concentrated target peaks with no spurious peaks in the background). Ablation experiments (Table 3) verify that removing DSDAM reduces IoU from 67.56% to 54.31% and Pd from 94.10% to 75.24%, directly demonstrating that this dynamic mechanism is the key to enhancing the targeting of feature extraction.

2. Core-Priority Loss Addresses the Problem of “Core Contribution Dilution”

Methods such as EGPNet adopt uniform weight losses (e.g., Dice and IoU losses) without considering the energy distribution characteristic of infrared small targets—strong center and weak edges. This results in a failure to prioritize the optimization of prediction errors in the core region, while edge noise instead interferes with model learning (e.g., edge artifacts of EGPNet and other methods in Figure 7). CECP-Loss assigns high weights to the target core region via “geometric center anchoring + Gaussian decay weight”, forcing the model to prioritize the optimization of energy-concentrated regions. Ablation experiments (Table 4) show that compared with the traditional Dice loss, CECP-Loss improves IoU by 0.55%, Pd by 1.15%, and reduces Fa by 0.19%. Transferring it to MSHNet (Table 5) increases IoU from 67.16% to 67.39%, demonstrating the generalizability and effectiveness of the core focusing of this loss function.

## 5. Conclusions

Infrared small-target segmentation (IRSTS) still faces two key technical challenges: the lack of contextual correlation in feature extraction, and the insufficient sensitivity of loss functions to target intrinsic characteristics. To solve these problems, this study proposes an infrared small-target segmentation framework based on morphological attention and energy core loss. The DSDAM adopts a parameterized strategy of “initial localization–offset deformation–precise sampling” to differentiate and emphasize the target’s core region and key background cues, thereby enhancing the target orientation of cross-layer feature fusion. CECP-Loss incorporates the energy prior distribution of infrared small targets (i.e., “stronger at the core and weaker at the edges”), effectively solving the “dilution of core contributions by edges” often observed in traditional loss functions. Experimental validation on public datasets such as IRSTD-1K and NUDT-SIRST reveals that the detection metrics (IoU, Pd, and Fa) of the proposed method are superior to those of existing mainstream methods; specifically, its IoU on the NUDT-SIRST dataset reaches 91.87%. These results fully confirm the synergistic effectiveness of the dynamic morphologically adaptive deformable attention module (DSDAM) and energy-aware loss (CECP-Loss), providing a better solution for IRSTS.

Aiming at the missed detection problem of small targets by the proposed method in ultra-low signal-to-noise ratio (SNR) scenarios, we will subsequently combine noise modeling and an adaptive energy enhancement module to further improve the robustness of the method in extreme scenarios with strong clutter and low SNR.

## Figures and Tables

**Table 1:** Comparative experimental results of average metrics on the NUDT-SIRST dataset. The best results are marked in red and bold, and the sub-best results in blue.

Method	IoU	P_d_	F_a_	Params (M)	FPS
ACLnet [47]	61.78	91.32	36.36	1.44	78.2
ISNet [44]	67.86	92.59	34.65	0.96	89.5
AGPCNet [48]	87.53	97.63	10.84	12.35	65.7
DNA-Net [45]	79.98	96.93	12.78	4.69	52.3
MSHNet [21]	80.55	97.99	11.77	4.07	58.6
EGPNet [33]	89.79	98.65	10.15	3.52	62.1
IRSTS_Unet (Ours)	**91.87**	**98.72**	**8.207**	4.12	63.8

**Table 2:** Comparative experimental results of average metrics on the IRSTD-1K dataset. The best results are marked in red and bold, and the sub-best results in blue.

Method	IoU	P_d_	F_a_
ACLnet [47]	62.03	91.75	42.46
ISNet [44]	62.88	92.59	27.92
AGPCNet [48]	56.02	91.50	17.10
DNA-Net [45]	65.71	91.84	17.61
MSHNet [21]	67.16	93.88	15.03
EGPNet [33]	66.62	93.95	24.20
IRSTS_Unet (Ours)	**67.56**	**94.10**	**15.02**

**Table 3:** Comparative experimental results of average metrics on the IRSTD-1K dataset. The best results are marked in red and bold, and the sub-best results in blue.

Method	IoU	P_d_	F_a_
ACLnet [47]	42.35	78.62	52.17
ISNet [44]	48.76	82.15	47.32
AGPCNet [48]	55.29	88.74	22.65
DNA-Net [45]	53.18	87.53	24.19
MSHNet [21]	54.62	88.16	23.57
EGPNet [33]	57.73	90.12	20.58
IRSTS_Unet (Ours)	**59.87**	**92.05**	**18.12**

**Table 4:** Comparative ablation experiment results of average metrics on the IRSTD-1K dataset.

Method	IoU	P_d_	F_a_
IRSTS-Unet-DSDAM + CECP-Loss	54.31	75.24	30.84
IRSTS-Unet + DSDAM + CECP-Loss	67.56	94.10	15.02

**Table 5:** Comparative ablation experiment results of average metrics on the IRSTD-1K dataset.

Method	IoU	P_d_	F_a_
IRSTS-Unet + DSDAM + Dice	67.01	92.95	15.21
IRSTS-Unet + DSDAM + CECP-Loss	67.56	94.10	15.02

**Table 6:** Comparative ablation experiment results of average metrics on the IRSTD-1K dataset.

Method	IoU	P_d_	F_a_
MSHNet [21]	67.16	93.88	15.03
MSHNet + Dice	65.16	92.18	14.12
MSHNet + CECP-Loss	67.39	94.01	15.03

## Data Availability

The data presented in this study are openly available in IRSTD-Unet at https://github.com/baoyu10/IRSTD-Unet (accessed on 1 January 2026).
